# Achieving normal pulmonary function following tracheoplasty in infancy

**DOI:** 10.1093/icvts/ivae152

**Published:** 2024-09-02

**Authors:** Ciara Harrison, Jo Harrison, Tyson A Fricke, Igor E Konstantinov

**Affiliations:** Department of Respiratory Medicine, Royal Children’s Hospital, Melbourne, Australia; Department of Cardiothoracic Surgery, Royal Children’s Hospital, Melbourne, Australia; Department of Respiratory Medicine, Royal Children’s Hospital, Melbourne, Australia; Department of Cardiothoracic Surgery, Royal Children’s Hospital, Melbourne, Australia; Department of Respiratory Medicine, Royal Children’s Hospital, Melbourne, Australia; Department of Cardiothoracic Surgery, Royal Children’s Hospital, Melbourne, Australia; Department of Paediatrics, University of Melbourne, Melbourne, Australia; Heart Research Group, Murdoch Children’s Research Institute, Melbourne, Australia; Melbourne Centre for Cardiovascular Genomics and Regenerative Medicine, Melbourne, Australia

**Keywords:** Trachea, Tracheal stenosis, Slide tracheoplasty

## Abstract

Infant long-segment congenital tracheal stenosis (LTS) is rare and presents a challenging clinical scenario. We describe the management of a child who required extracorporeal membrane oxygenation following a respiratory arrest and underwent slide tracheoplasty in infancy for severe LTS and required repeated bronchoscopic reinterventions for recurrent tracheal granulations. At 9 years of age, the child has normal pulmonary function testing and a normal exercise tolerance.

## INTRODUCTION

Management of long-segment congenital tracheal stenosis (LTS) in severely symptomatic infants is challenging. Herein, we describe perioperative management of a child, who underwent tracheoplasty in infancy for severe LTS, which resulted in normal pulmonary function test and normal exercise tolerance at 9 years of age. The patient’s family provided consent for the publication of this report.

**Figure 1:**
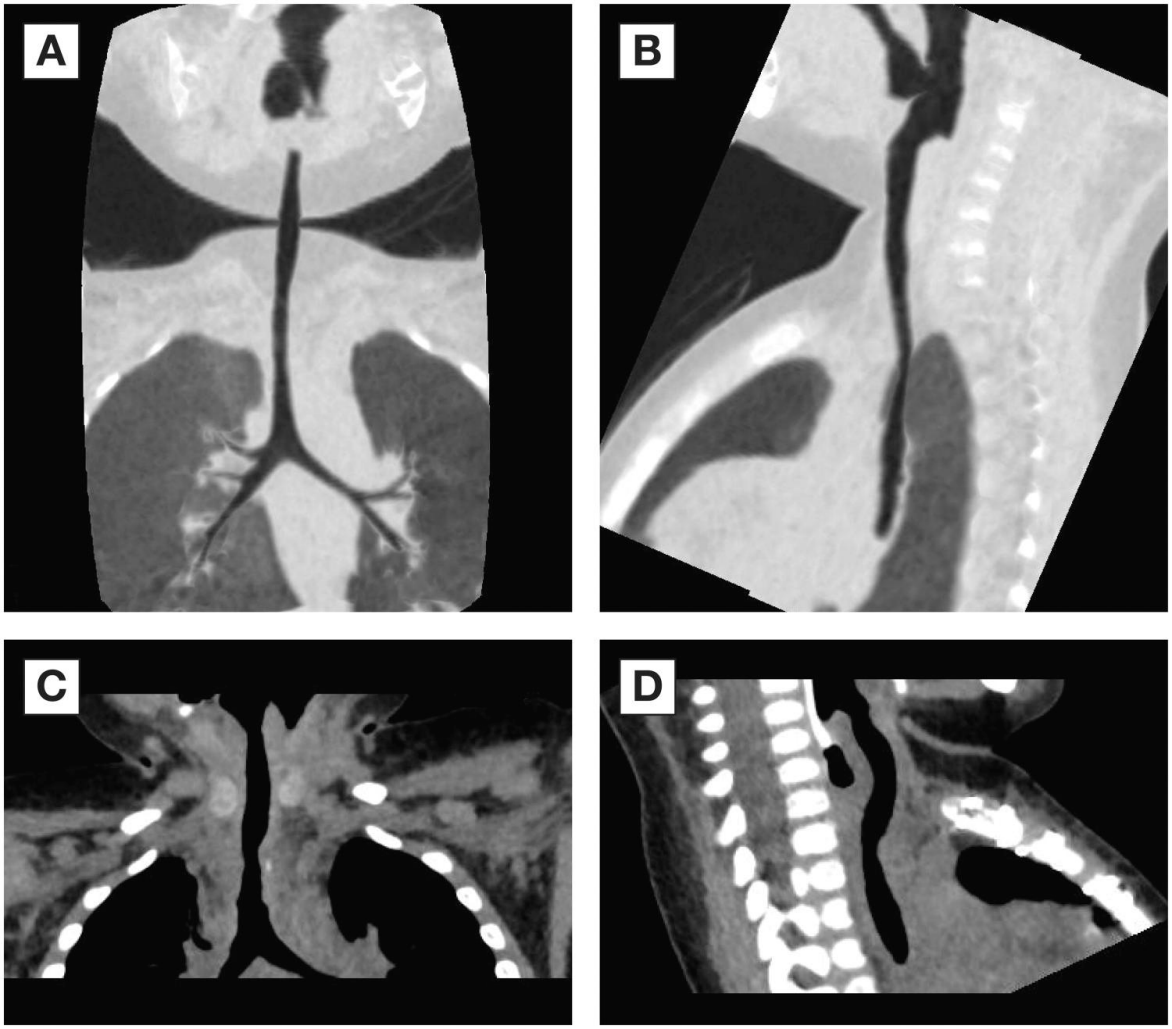
Preoperative and postoperative views. Preoperative coronal (**A**) and sagittal (**B**) views and postoperative coronal (**C**) and sagittal (**D**) views.

An 11-month-old boy with known LTS (Fig. 1) was urgently transferred to the Royal Children’s Hospital from an outside institution with stridor in the setting of an acute respiratory tract infection and respiratory distress. He required emergent intubation. Yet, an adequate ventilation could not be achieved as the lungs could not be deflated and had to be deflated manually. He had a respiratory arrest and required emergent cannulation for extracorporeal membrane oxygenation (ECMO), which was done via midline sternotomy. Bronchoscopy confirmed severe tracheal stenosis with complete tracheal rings in the lower two-thirds of the trachea. The patient required thorough planning and complex perioperative management. He underwent slide tracheoplasty (STP) on the same day. STP of lower two-thirds of the trachea was performed and autologous pericardium was placed between the trachea and great vessels as previously described [1].

Two weeks post-surgery, he developed increasing obstructive airway symptoms. Flexible bronchoscopy demonstrated tracheal narrowing due to significant granulation tissue formation, which was treated by balloon dilation. Due to recurrent episodes of severe airway obstruction, the patient required weekly balloon dilations for 6 weeks due to persistent intratracheal granulations. At 2 months, a custom-made, biodegradable, self-expanding poly-dioxanone stent (ELLA-CS, Hradec Kralove, Czech Republic) was then deployed in his trachea with good effect. Due to granulation development proximal to the stent, balloon dilatation and placement of 2nd stent of the same type was done 2 weeks later. Both stents slowly migrated distally and were removed in a month after placement. Computed tomography (CT) scan was then done at 4 months after tracheoplasty demonstrated a stable trachea. Yet, within the next 10 days, he developed severe tracheal obstruction due to granulations and required placement of another stent of the same type. Due to persistent significant granulation tissues coming through the stent, he underwent several balloon dilatations and immunosuppressive therapy began at 6 months after STP. The patient received budesonide for 6 months and 3 cycles (2 months each with 1 month in between the cycles) of sirolimus. During each cycle of sirolimus, the patient received prophylactic antibiotic coverage with sulfamethoxazole/trimethoprim (Table 1). The last biodegradable stent was placed at 1 year after surgery and immediately before the last cycle of sirolimus was commenced. Single doses of dexamethasone were given at times of airway intervention. The granulation tissue formation subsided, and he only required 2 balloon dilatations during the last cycle of sirolimus. At 15 months after STP, he underwent a final bronchoscopy and has required no further procedures. Thus, during a 15-month period after slide tracheoplasty, the child required placement of 4 biodegradable tracheal stents and 23 balloon dilatations for persistent intratracheal granulations.

**Table 1:** Pharmacological management of recurrent tracheal granulations

Drug	Dose	When given
Dexamethasone	0.6 mg/kg intravenously	During bronchoscopies
Sirolimus	2 mg/m^2^ oral daily aiming levels 5–10 ng/ml with sulfamethoxazole/trimethoprim prophylaxis	Start 6 months after tracheoplasty. Duration: 2 months
		Start 9 months after tracheoplasty. Duration: 2 months
		Start 13 months after tracheoplasty. Duration: 2 months
Budesonide	1 mg/2 ml nebulized twice a day.	Start: 6 months after tracheoplasty. Duration: 9 months

At the age of 9 years, he had normal exercise tolerance and normal lung function. Forced expiratory volume in 1 s (FEV_1_) was 1.36 l (82% predicted), forced vital capacity (FVC) is 1.69 l (89.5% predicted) and the FEV_1_/FVC ratio was 81%.

## DISCUSSION

Long-segment tracheal stenosis due to complete tracheal rings is a rare, but life-threatening congenital anomaly, which often requires surgical intervention. STP has emerged as the technique of choice for LTS due to improved outcomes with this technique [2]. Tracheal restenosis and airway obstruction due to granulation tissue are well-described complications of STP, which often require airway interventions including balloon dilation and stent placement. Beeman *et al.* [3] described the outcomes of 150 patients who underwent STP for LTS between 1995 and 2017 and reported that 81 (54%) of the patients required airway intervention after tracheoplasty with 19% requiring tracheal stenting. Another large case series also reported high rates of airway reintervention with 34 of 80 patients (42%) in their series requiring reintervention in the postoperative period [4]. Several risk factors, including preoperative ECMO, have been found to be associated with poorer outcomes following STP as well as the need for postoperative airway intervention [3, 4]. Granulation tissue is a common and well-recognized postoperative complication in these patients and is associated with need for prolonged mechanical ventilation, multiple airway interventions and mortality [5].

While appropriate tracheal growth postoperatively has been demonstrated, data regarding functional outcomes studies after STP are limited. A study from our institution demonstrated that an obstructive pulmonary flow pattern is common in patients who underwent STP in infancy at the time of repair of pulmonary artery sling [5]. Thus, spirometry performed a median of 9 years post tracheal surgery in 9 patients demonstrated moderate–severe airway obstruction. Although the exact causes of severe persistent granulations after tracheoplasty in infancy are not clear, this case demonstrates that biodegradable stenting in combination with controlled immunosuppressive therapy can result in excellent long-term outcomes.

## Data Availability

The data underlying this article cannot be shared publicly due to the privacy of the individual who participated in the study.
